# Risk for contralateral breast cancer among carriers of the CHEK2^*^1100delC mutation in the WECARE Study

**DOI:** 10.1038/sj.bjc.6604228

**Published:** 2008-02-05

**Authors:** L Mellemkjær, C Dahl, J H Olsen, L Bertelsen, P Guldberg, J Christensen, A-L Børresen-Dale, M Stovall, B Langholz, L Bernstein, C F Lynch, K E Malone, R W Haile, M Andersson, D C Thomas, P Concannon, M Capanu, J D Boice, The WECARE Study Collaborative Group, J L Bernstein

**Affiliations:** 1 Institute of Cancer Epidemiology, Danish Cancer Society, Strandboulevarden 49, DK-2100 Copenhagen, Denmark; 2 Institute of Cancer Biology, Danish Cancer Society, Strandboulevarden 49, DK-2100 Copenhagen, Denmark; 3 Department of Genetics, Institute for Cancer Research, The Norwegian Radium Hospital, N-0310 Oslo, Norway; 4 Faculty of Medicine, University of Oslo, Postboks 1171 Blindern, N0318 Oslo, Norway; 5 Department of Radiation Physics, Unit 544, The University of Texas MD Anderson Cancer Center, 1515 Holcombe, Houston, TX 77030, USA; 6 Department of Preventive Medicine, University of Southern California, 1441 Eastlake Avenue USC/Norris Comprehensive Cancer Center NOR-4435, Los Angeles, CA 90089-9175, USA; 7 Department of Epidemiology, University of Iowa, 200 Hawkins Drive, Iowa City, IA 52242, USA; 8 Division of Public Health Sciences, Fred Hutchinson Cancer Research Center, 1100 Fairview Avenue North, M4-B814, Seattle, WA 98109, USA; 9 Department of Oncology, The Finsen Center, Rigshospitalet, University of Copenhagen, Blegdamsvej 9, Copenhagen DK-2100, Denmark; 10 Department of Biochemistry and Molecular Genetics, University of Virginia, PO Box 800733, Charlottesville, VA 22908, USA; 11 Department of Epidemiology and Biostatistics, Memorial Sloan-Kettering Cancer Center, 307 East 63rd Street, 3rd Floor, New York, NY 10021, USA; 12 International Epidemiology Institute, 1455 Research Blvd, Suite 550, Rockville, MD 20850, USA; 13 Department of Medicine, Vanderbilt University Medical Center, 21st Avenue South and Garland Avenue, Nashville, TN 37232, USA

**Keywords:** asynchronous contralateral breast cancer, CHEK2^*^1100delC mutation, genes, radiation therapy, chemotherapy

## Abstract

The protein encoded by the *CHEK2* gene is involved in cellular repair of DNA damage. The truncating mutation, CHEK2^*^1100delC, seems to increase the risk for breast cancer. We investigated whether the CHEK2^*^1100delC mutation carrier status increases the risk for asynchronous contralateral breast cancer (CBC) and whether it interacts with radiation therapy (RT) or chemotherapy in regard to CBC risk. The germline mutation frequency was assessed in 708 women with CBC and 1395 women with unilateral breast cancer (UBC) in the Women's Environment, Cancer and Radiation Epidemiology (WECARE) Study whose first primary breast cancer was diagnosed before age 55 years and during 1985–1999. Seven women with CBC (1.0%) and 10 women with UBC (0.7%) were CHEK2^*^1100delC variant carriers (rate ratio (RR)=1.8, 95% confidence interval (CI)=0.6–5.4 for CBC *vs* UBC). Carriers who received RT for their first breast cancer, compared with non-carriers not treated with RT, had an RR of developing CBC of 2.6 (95% CI=0.8–8.7). We found no significant associations between the CHEK2^*^1100delC mutation and CBC overall or among those treated with RT. However, the sampling variability was such that modest increases in risk could not be excluded. Nonetheless, because this is a rare mutation, it is unlikely to explain a major fraction of CBC in the population.

The *CHEK2* gene codes for a protein that is involved in cell-cycle control and DNA repair. A protein-truncating mutation in the gene, 1100delC, was associated with a two-fold increase in breast cancer risk in the CHEK2 Breast Cancer Case–Control Consortium pooling project, which comprised 10 860 breast cancer cases and 9065 controls from 10 studies in five countries (The CHEK2 Breast Cancer Case–Control Consortium, 2004). The prevalence of the CHEK2^*^1100delC variant among women with and without breast cancer varies among countries (Meijers-Heijboer *et al*, 2002; Vahteristo *et al*, 2002; Osorio *et al*, 2004; Huzarski *et al*, 2005; Kilpivaara *et al*, 2005; Bernstein *et al*, 2006; Weischer *et al*, 2007). A higher prevalence of the mutation has been found among patients with bilateral breast cancer (Broeks *et al*, 2004; Kilpivaara *et al*, 2005) and among patients with a family history of breast cancer (Meijers-Heijboer *et al*, 2002; Vahteristo *et al*, 2002; Friedrichsen *et al*, 2004). Carriers of the CHEK2^*^1100delC mutation are hypothesised to have an increased sensitivity to exposures such as radiation therapy (RT) and chemotherapy that cause DNA double-strand breaks, as the CHEK2 regulates cell-cycle checkpoint response pathways that respond to such damage (Nevanlinna and Bartek, 2006). We investigated the risk for contralateral breast cancer (CBC) associated with the CHEK2^*^1100delC germline mutation among approximately 2100 women with breast cancer who were cases and controls in the multicentre Women's Environment, Cancer and Radiation Epidemiology (WECARE) Study (Bernstein *et al*, 2004). In addition, we explored the joint effect of being a carrier of mutated *CHEK2* and having received RT or chemotherapy for breast cancer.

## MATERIALS AND METHODS

### Study population

Cases in this study were women with asynchronous CBC and controls were women with unilateral breast cancer (UBC) obtained from the WECARE Study (Bernstein *et al*, 2004). The participants were identified through five population-based cancer registries: four in the United States (Surveillance Epidemiology and End Results (SEER) registries in Iowa, Seattle, Los Angeles County and Orange County/San Diego) and one in Denmark (Danish Breast Cancer Cooperative Group (DBCG) registry supplemented by data from the Danish Cancer Registry). Case women were eligible for the study if they were under age 55 years at diagnosis of their first invasive breast cancer and were diagnosed with a second primary breast cancer in the contralateral breast (invasive or *in situ)* at least 1 year later, while residing in the same reporting area at the time of diagnosis of both cancers with no history of a prior cancer diagnosis or an intervening cancer diagnosis between their first and second primary breast cancers. All first primary breast cancers were invasive without distant metastases and were diagnosed between 1 January 1985 and 31 December 1999. The second primary breast cancer had to have been diagnosed no later than 31 December 2001. All women were alive at the time of contact. Control women had to fulfil the same eligibility criteria as cases except that they had no CBC during the interval between the matched case woman's first and second breast cancers (‘at-risk’ period) and had no prophylactic mastectomy during the ‘at-risk’ period. The reference date for case women was the date of diagnosis of their second primary breast cancer, whereas the corresponding reference date for control women was defined as their date of diagnosis of primary breast cancer plus the ‘at-risk’ period for their matched case. Two UBC controls were individually matched to each CBC case on year of birth (5-year strata), year of diagnosis (4-year strata), registry region and race, and counter-matched on RT (as designated by the cancer registry), that is, each triplet comprised two women who had received RT and one woman who had not. Counter-matching was used to improve the statistical efficiency of the study design (Bernstein *et al*, 2004).

Characteristics of the first and second breast cancers were abstracted from medical records and cancer registry files. Medical records were retrieved to obtain detailed information on the treatment of the first breast cancer (RT, chemotherapy and hormonal therapy). By the use of methods previously described (Bernstein *et al*, 2004), the mean individual radiation dose to the contralateral breast was estimated as 1.29 Gy (range: 0.03–4.68 Gy) (estimation carried out for 85% of those who received RT). Among women treated with chemotherapy, 54% had received CMF, CF or CM (C, cyclophosphamide; M methotrexate; F, fluorouracil), 23% had received CAF, CEF, CMAF, CAM, CMEF or AC (A, adriamycin; E, epirubicine) and 23% had received other or unknown regimens. All the women were interviewed by telephone using a structured questionnaire to obtain information on known breast cancer risk factors. In addition, for the small subset of women for whom medical records did not give information on chemotherapy (7%) and/or hormonal therapy (10%), treatment information was obtained at the interview. Blood samples were drawn by study phlebotomists. Study participants were genotyped for mutations in *BRCA1* and *BRCA2* genes as previously described (Begg *et al*, 2008). All participants provided written informed consent before enrolment into the WECARE Study, and the research protocol was approved by the respective Institutional Review Boards and the ethical committee system in Denmark.

Among 998 CBC patients (cases) and 2112 UBC controls who were eligible and approached to participate in the study, 708 (71%) cases and 1399 (66%) controls were enrolled. Matching was successfully carried out for 694 triplets (matching criteria were relaxed on region or race/ethnicity for 17 triplets) and 11 case–control pairs, whereas three cases had no matched controls. All 708 cases and 1395 of the controls (99.7%) were genotyped for CHEK2^*^1100delC (four controls excluded).

### Genotyping of CHEK2^*^1100delC

**Figure 1:**
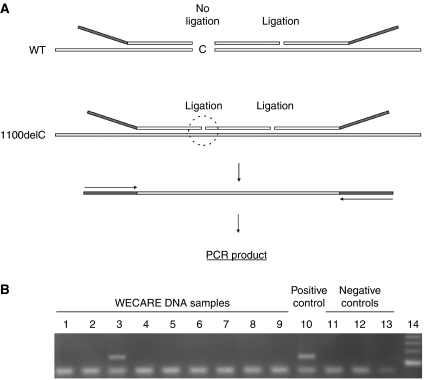
Detection of CHEK2^*^1100delC by oligonucleotide ligation. (**A**) Schematic of the method. Total genomic DNA is incubated with three oligonucleotides. On the CHEK2^*^1100delC sequence, the oligonucleotides hybridise in juxtaposition on the target sequence and can be joined by ligation and subsequently amplified by PCR. On the wild-type *CHEK2* sequence, two of the oligonucleotides are separated by one base (C) and cannot be ligated into an amplifiable probe. (**B**) Analysis of WECARE samples. The controls include DNA samples with (lane 10) and without (lane 11) CHEK2^*^1100delC, a ligation control without DNA (lane 12), and a negative PCR control (lane 13). Lane 14, 100 bp ladder.

For detection of CHEK2^*^1100delC, we developed an assay based on ligation-dependent amplification (Figure 1). The sequences of the oligonucleotides were 5′-[TATGTAAAACGACGGCCAGT]-TGGCAAGTTCAACATTATTCCCTTTTGTACTGAATTTTAGATTA-3′, 5′-TGATTTTGGGCACTCCAAGATTTTGGG-3′ and 5′-AGAGACCTCTCTCATGAGAACCTTATGTGGAACC-[ACCCAATTCGCCCTATAATA]-3′; the latter two oligonucleotides were 5′-phosphorylated. Approximately 50 ng of genomic DNA in 5 *μ*l TE buffer was incubated at 98°C for 10 min and then cooled at 25°C. MLPA buffer (1.5 *μ*l; MRC-Holland, Amsterdam, the Netherlands) and 2 fmol of each oligonucleotide were added, and the samples were then heated at 95°C for 1 min and incubated at 37°C for 16 h in a total volume of 8 *μ*l. Ligation of the hybridised oligonucleotides was achieved by the addition of 32 *μ*l of a ligase mix containing 3 *μ*l ligase buffer A (MRC-Holland), 3 *μ*l ligase buffer B (MRC-Holland), 1 U Taq DNA ligase (New England Biolabs, Beverly, MA, USA) and incubation at 54°C for 15 min. Ligation products were amplified with primers 5′-TATGTAAAACGACGGCCAGT-3′ and 5′-TATTATAGGGCGAATTGGGT-3′. Hybridisation, ligation and PCR were performed in 96-well plates, which contained two positive controls (DNA with CHEK2^*^1100delC), two negative controls (DNA without CHEK2^*^1100delC) and two ligation controls (without DNA). The PCR products were resolved by agarose gel electrophoresis.

The reliability of the ligation assay was confirmed by re-analysis of 248 DNA samples from WECARE Study participants using denaturing gradient gel electrophoresis (DGGE), as described previously (Bartkova *et al*, 2004). We found complete concordance between the results obtained with these two methods (3 out of 248 samples screened were positive for mutations). The remaining 1855 samples were then analysed with the ligation assay. All positive results were reconfirmed by DGGE analysis or with the ligation assay for samples where long-range amplification was unsuccessful.

### Statistical methods

Rate ratios (RRs) and corresponding 95% confidence intervals (CIs) were estimated by univariate conditional logistic regression models accounting for the counter-matched design (details described previously; Bernstein *et al*, 2004). Multivariable models were adjusted for exact age at diagnosis of the first primary breast cancer, age at menarche (<13/⩾13 years), menopausal status at reference date (premenopausal/age at menopause <45 years/age at menopause ⩾45 years), number of full-term pregnancies (0, 1, 2, 3, ⩾4), family history of breast cancer among first-degree relatives (yes/no), lobular histology of the first primary (yes/no), stage of first breast cancer (local/regional), RT (yes/no), chemotherapy (yes/no) and hormonal therapy (yes/no). As results of the univariate and multivariable models were quite similar, we present RRs from the univariate models. In addition, we present RRs from models adjusted for *BRCA1/2* carrier status (deleterious *BRCA1/2* mutations yes/no/unknown). The TPHREG procedure in SAS release 9.1 (SAS Institute Inc., Cary, NC, USA) was used for the statistical analyses. Further information about the SAS programming used for counter-matched WECARE data is provided in the appendix of Bernstein *et al* (2004).

## RESULTS

The mean length of time since first breast cancer diagnosis was 5.0 years (range: 1–15.6 years) for the total study population. Seven (1.0%) carriers of the CHEK2^*^1100delC germline mutation were found among the 708 women with CBC, whereas 10 (0.7%) carriers were found among the 1395 women with UBC. Characteristics of carriers and non-carriers among cases and controls are shown in Table 1.

The association between being carrier of the CHEK2^*^1100delC mutation and risk of developing CBC was not statistically significant (RR=1.7; 95% CI=0.6–5.1) (Table 2). Compared to women who did not carry the mutation and who did not receive RT for their first breast cancer, carriers who received RT as treatment had an RR of CBC of 2.6 (95% CI=0.8–8.4). The RR of CBC among mutation carriers treated with chemotherapy was 1.3 (95% CI=0.3–6.2) compared with non-carriers who did not receive chemotherapy. We had 90% statistical power at two-sided *P*<0.05 to exclude a RR of 6.0 for CBC associated with the CHEK2^*^1100delC mutation overall, a RR of 6.9 for the combination of the mutation and RT and a RR of 13.1 for the combination of the mutation and chemotherapy. The RR for the CHEK2^*^1100delC mutation and CBC overall was largely unchanged after the adjustment for *BRCA1/2* carrier status (RR=1.8; 95% CI=0.6–5.4) as were the RRs for the mutation in combination with RT or chemotherapy (Table 2).

Results of analyses restricted to non-Hispanic whites (649 women with CBC and 1286 women with UBC) were similar to those for all study subjects combined.

## DISCUSSION

In this population-based multicentre study, we found a low prevalence of CHEK2^*^1100delC mutation carriers among 708 women with CBC (1.0%) and 1395 women with UBC (0.7%). Several studies have shown higher frequencies (ranging from 1.7 to 14.3%) of mutation among women with bilateral breast cancer (Oldenburg *et al*, 2003; Broeks *et al*, 2004; Kilpivaara *et al*, 2005; Chekmariova *et al*, 2006; Kwiatkowska *et al*, 2006); however, none of these were population-based. Two of these studies reported that the frequency of mutation was significantly increased among women with bilateral breast cancer compared to women with UBC (Broeks *et al*, 2004; Kilpivaara *et al*, 2005). Also, a small but prospective study reported a significant increase in risk for CBC among mutation carriers (de Bock *et al*, 2004). Three previous studies showing a high carrier prevalence among bilateral breast cancer cases were from the North European countries, the Netherlands (Oldenburg *et al*, 2003; Broeks *et al*, 2004) and Finland (Kilpivaara *et al*, 2005). Although the carrier prevalence among Danish WECARE Study participants is low relative to these prior reports, the prevalence among Danish participants with UBC is similar to that observed in a previous Danish study (Weischer *et al*, 2007). Differences in frequencies across studies may also be due to differences in study selection criteria, for example, restriction to certain ages at the onset of breast cancer and/or to subjects with family history defined in various ways. Only one of the previous studies on bilateral breast cancer was restricted to non-*BRCA1/2* mutation cases (Oldenburg *et al*, 2003). In contrast to all the previous studies on bilateral breast cancer, which were based on cases from cancer hospitals or genetics clinics, our study is the first that is population-based.

The CHEK2^*^1100delC allele is hypothesised to interact with environmental factors to promote breast tumorigenesis. Ionising radiation is of particular interest because it induces DNA damage, and the protein of the *CHEK2* gene is a kinase that plays a role in response to such damage by delaying cell-cycle progression to facilitate DNA repair or even induce cell death (Nevanlinna and Bartek, 2006). The germline mutation 1100delC truncates the CHEK2 protein, thereby abolishing the kinase function (Wu *et al*, 2001). Epidemiological evidence to substantiate increased radiosensitivity among carriers is, however, sparse (Bernstein *et al*, 2006; Broeks *et al*, 2007). Also, although a study conducted in Belgium suggested that breast cancer patients with a CHEK2^*^1100delC mutation are, in general, not characterised by a distinct enhanced chromosomal radiosensitivity, this conclusion was based on only four breast cancer cases with the mutation who were compared with healthy controls without the mutation (Baeyens *et al*, 2005). Our result, although not statistically significant, suggests that mutation carriers may have increased radiosensitivity.

The WECARE Study was designed to investigate interactions between genes and exposures that cause DNA double-strand breaks in a study population enriched with hereditary breast cancer cases, owing to the restriction to early-onset breast cancer, and a population heavily exposed to ionising radiation and chemotherapeutic drugs. Detailed information on RT and chemotherapy was obtained by a thorough review of medical records. As DNA samples from WECARE Study participants have also been screened for mutations in *BRCA1* and *BRCA2* genes, we were able to adjust the analyses for *BRCA* carrier status. Patients included in the study survived varying lengths of time from reference date to interview; however, cases and controls within each triplet survived approximately the same length of time. The prevalence of the mutation may have been affected if carriers of the CHEK2^*^1100delC mutation had a worse survival than non-carriers. Unfortunately, we could not address this question, as we had no information on carrier status among the non-survivors who were not included in the study. Previous studies have shown a worse recurrence-free survival (Schmidt *et al*, 2007) and distant metastases-free survival (de Bock *et al*, 2004) among carriers compared to non-carriers, but overall survival did not differ by carrier status (de Bock *et al*, 2004).

Notably, we found a low carrier frequency for the CHEK2^*^1100delC mutation in this large population-based sample of early-onset cases of CBC and UBC. The small number of carriers reduced our ability to study the effects of the mutation on the development of CBC; and accordingly, we could not exclude the possibility that the CHEK2^*^1100delC mutation is associated with modest increases in risk. In contrast, the rarity of the CHEK2^*^1100delC mutation indicates that it plays a rather limited role in the aetiology of CBC in the population.

## Figures and Tables

**Table 1:** Frequency of CHEK2^*^1100delC carrier status among women with asynchronous CBC and UBC in the WECARE Study according to age at first breast cancer, race, study centre, family history of breast cancer and estrogen receptor status of first breast cancer

	**Asynchronous CBC (*n*=708)**		**UBC (*n*=1395)**	
	**Carrier**	**Non-carrier**	**Carrier**	**Non-carrier**
All (*n* and %)	7 (1.0)	701 (99.0)	10 (0.7)	1385 (99.3)
				
*Age at first breast cancer (n* *and* %)				
<45 years	2 (0.7)	289 (99.3)	6 (1.1)	562 (98.9)
⩾45 years	5 (1.2)	412 (98.8)	4 (0.5)	823 (99.5)
				
*Race (n* *and* %)				
Non-Hispanic white	7 (1.1)	642 (98.9)	10 (0.8)	1276 (99.2)
Hispanic white	0 (—)	24 (100.0)	0 (—)	46 (100.0)
Black	0 (—)	21 (100.0)	0 (—)	39 (100.0)
Asian	0 (—)	13 (100.0)	0 (—)	22 (100.0)
Other	0 (—)	1 (100.0)	0 (—)	2 (100.0)
				
*Study centre (n* *and* %)				
Los Angeles County, CA	1 (0.5)	198 (99.5)	1 (0.3)	390 (99.7)
Seattle	1 (1.0)	98 (99.0)	0 (—)	193 (100.0)
Iowa	3 (2.7)	110 (97.3)	4 (1.8)	224 (98.2)
Orange County/San Diego, CA	0 (—)	118 (100.0)	2 (0.9)	227 (99.1)
Denmark	2 (1.1)	177 (98.9)	3 (0.8)	351 (99.2)
				
*Family history of breast cancer (n* *and* %)				
At least one first-degree relative with breast cancer^a^	3 (1.3)	223 (98.7)	5 (1.7)	284 (98.3)
At least one second-degree relative with breast cancer^a^	3 (1.1)	274 (98.9)	2 (0.5)	409 (99.5)
At least one relative with bilateral breast cancer^a^	1 (6.7)	14 (93.3)	0 (—)	18 (100.0)
Unknown family history, adopted	0 (—)	11 (100.0)	0 (—)	26 (100.0)
				
*Estrogen receptor status, first breast cancer (n* *and* %)				
Positive	4 (1.2)	334 (98.8)	4 (0.5)	739 (99.5)
Negative	3 (1.6)	190 (98.4)	3 (0.9)	335 (99.1)
Other or unknown	0 (—)	177 (100.0)	3 (1.0)	311 (99.0)

CBC=contralateral breast cancer; WECARE=Women's Environment, Cancer and Radiation Epidemiology; UBC=unilateral breast cancer.

a Women may be in more than one of these categories.

**Table 2:** Risk for asynchronous CBC associated with CHEK2^*^1100delC mutation carrier status overall and in combination with RT or chemotherapy for the first breast cancer

			**Univariate^b^**		**Adjusted^c^**	
	**No. with asynchronous CBC (*n*=705^a^)**	**No. with UBC (*n*=1395)**	**RR**	**95% CI**	**RR**	**95% CI**
*Mutation*						
No	698	1385	1.00	—	1.00	—
Yes	7	10	1.7	0.6–5.1	1.8	0.6–5.4
						
*Mutation* × *RT*						
No × no	359	263	1.00	—	1.00	—
No × yes	339	1122	1.1	0.9–1.3	1.0	0.9–1.2
Yes × no	2	2	0.6	0.1–4.2	0.7	0.1–5.4
Yes × yes	5	8	2.6	0.8–8.4	2.6	0.8–8.7
						
*Mutation* × *chemotherapy*						
No × no	381	622	1.00	—	1.00	—
No × yes	317	763	0.6	0.5–0.7	0.6	0.5–0.7
Yes × no	4	5	1.4	0.3–6.3	1.6	0.4–7.6
Yes × yes	3	5	1.3	0.3–6.2	1.2	0.3–5.7

CBC=contralateral breast cancer; CI=confidence interval; RR=rate ratio; RT=radiation therapy; UBC=unilateral breast cancer.

a Three cases without matched controls excluded.

b RRs adjusted for counter-matching sampling.

c RRs adjusted for counter-matching sampling and *BRCA1/2* carrier status.

## Appendix

### CORPORATE AUTHORSHIP LIST

WECARE Study Collaborative Group for WECARE: *CHEK2^*^1100delC*

PI: Jørgen H Olsen, MD, DMSc; Co-investigators named on grant: Jonine L Bernstein, PhD, Anne-Lise Børresen-Dale, PhD, Åke Borg, PhD, Lisbeth Bertelsen, MD, Lene Mellemkjær, PhD, MSc, Per Guldberg, PhD.

Coordinating Center: Memorial Sloan-Kettering Cancer Center (New York, NY, USA) Jonine L Bernstein, PhD (WECARE Study PI), Xiaolin Liang, MD, MS (Informatics Specialist), Abigail Wolitzer, MSPH (Project Director); National Cancer Institute (Bethesda, MD, USA) Daniela Seminara, PhD, MPH (Program Officer).

Laboratories^*^: Danish Cancer Society (Copenhagen, Denmark) Per Guldberg, PhD; University of Southern California (Los Angeles, CA, USA) Robert W Haile, Dr PH (PI), Anh T Diep (Laboratory Director), Nianmin Zhou, MD (Laboratory Manager), Yong Liu, MD (Director of Blood Processing), Evgenia Ter-Karapetova (Supervisor of Biospecimen Processing), Andre Hernandez; Memorial Sloan-Kettering Cancer Center (New York, NY, USA) Irene Orlow, PhD (Laboratory Director, Biorepository).

Epidemiology/Data Collection Centers^*^: Danish Cancer Society (Copenhagen, Denmark) Jørgen H Olsen, MD, DMSc (PI), Lene Mellemkjær, PhD, MSc (Project Manager), Per Guldberg, PhD, Lisbeth Bertelsen, MD; University of Southern California (Los Angeles, CA, USA) Leslie Bernstein, PhD (PI), Laura Donnelly-Allen (Project Manager); University of Iowa (Iowa City, IA, USA) Charles F Lynch, MD, PhD (PI), Jeanne DeWall, MA (Project Manager); Fred Hutchinson Cancer Research Center (Seattle, WA, USA) Kathleen E Malone, PhD (PI), Noemi Epstein (Project Manager); University of California at Irvine (Irvine, CA, USA) Hoda Anton-Culver, PhD (PI), Joan Largent, PhD, MPH (Project Manager).

Radiation Measurement: University of Texas, MD Anderson Cancer Center (Houston, TX, USA) Marilyn Stovall, PhD (PI), Susan Smith, MPH (Quality Assurance Dosimetry Supervisor); New York University (New York, NY, USA) Roy E Shore, PhD, Dr PH (Epidemiologist); International Epidemiology Institute (Rockville, MD, USA) and Vanderbilt University (Nashville, TN, USA) John D Boice Jr, ScD (Consultant).

Biostatistics Core: University of Southern California (Los Angeles, CA, USA) Bryan M Langholz, PhD, Duncan C Thomas, PhD; Memorial Sloan-Kettering Cancer Center (New York, NY, USA) Colin Begg, PhD, Marinela Capanu, PhD; University of Southern Maine (Portland, ME, USA) W Douglas Thompson, PhD (PI).
